# *rac*-3-{4-[(Furan-2-ylmethyl­idene)­amino]-3-methyl-5-sulfanyl­idene-4,5-dihydro-1*H*-1,2,4-triazol-1-yl}-1,3-diphenyl­propan-1-one

**DOI:** 10.1107/S1600536811035185

**Published:** 2011-09-30

**Authors:** Wei Wang, Yan Gao, Wen-peng Wu, Chao Xu, Qing-lei Liu

**Affiliations:** a School of Perfume and Aroma Technology, Shanghai Institute of Technology, Shanghai 200235, People’s Republic of China; b School of Chemical Engineering, University of Science and Technology LiaoNing, Anshan 114051, People’s Republic of China

## Abstract

In the title mol­ecule, C_23_H_20_N_4_O_2_S, the triazole ring forms dihedral angles of 150.3 (2), 77.3 (2) and 77.6 (2)°, respectively, with the furan ring and the phenyl rings. The furan ring is almost perpendicular to the central phenyl ring, making a dihedral angle of 86.0 (3)°.

## Related literature

For the crystal structures of related 1,2,4-triazole-5(4*H*)-thione derivatives, see: Al-Tamimi *et al.* (2010 ▶); Fun *et al.* (2009 ▶); Gao *et al.* (2011 ▶); Tan *et al.* (2010 ▶); Wang *et al.* (2011 ▶); Zhao *et al.* (2010 ▶).
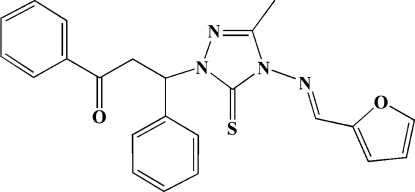


## Experimental

### 

#### Crystal data

C_23_H_20_N_4_O_2_S
*M* *_r_* = 416.50
Monoclinic, 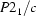

*a* = 8.154 (3) Å
*b* = 21.194 (6) Å
*c* = 12.878 (4) Å
β = 107.965 (5)°
*V* = 2117.0 (12) Å^3^
*Z* = 4
Mo *K*α radiation
μ = 0.18 mm^−1^
*T* = 113 K
0.24 × 0.20 × 0.12 mm

#### Data collection

Rigaku Saturn CCD area-detector diffractometer
Absorption correction: multi-scan (*CrystalClear*; Rigaku/MSC, 2005 ▶) *T*_min_ = 0.958, *T*_max_ = 0.979
26979 measured reflections
5030 independent reflections
4229 reflections with *I* > 2σ(*I*)
*R*_int_ = 0.043

#### Refinement

*R*[*F* ^2^ > 2σ(*F* ^2^)] = 0.040
*wR*(*F* ^2^) = 0.106
*S* = 1.07
5030 reflections
272 parameters
H-atom parameters constrained
Δρ_max_ = 0.33 e Å^−3^
Δρ_min_ = −0.18 e Å^−3^

### 

Data collection: *CrystalClear* (Rigaku/MSC, 2005 ▶); cell refinement: *CrystalClear*; data reduction: *CrystalClear*; program(s) used to solve structure: *SHELXS97* (Sheldrick, 2008 ▶); program(s) used to refine structure: *SHELXL97* (Sheldrick, 2008 ▶); molecular graphics: *SHELXTL* (Sheldrick, 2008 ▶); software used to prepare material for publication: *SHELXTL*.

## Supplementary Material

Crystal structure: contains datablock(s) global, I. DOI: 10.1107/S1600536811035185/zs2141sup1.cif

Structure factors: contains datablock(s) I. DOI: 10.1107/S1600536811035185/zs2141Isup2.hkl

Supplementary material file. DOI: 10.1107/S1600536811035185/zs2141Isup3.cml

Additional supplementary materials:  crystallographic information; 3D view; checkCIF report

## Figures and Tables

**Fig. 1.**
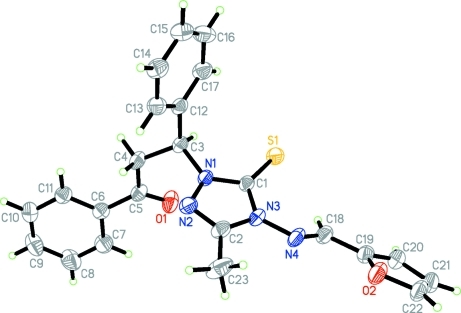
A view of the molecule of the title compound showing the atom-labelling scheme. Displacement ellipsoids are drawn at the 60% probability level.

## supplementary crystallographic information

### Comment

In a continuation of structural studies by our group (Wang *et al.*, 2011) of Mannich base derivatives synthesized by reactions of amino heterocycles and aromatic aldehydes, we present here the crystal structure of the title compound, C_23_H_20_N_4_O_2_S.

The bond lengths and angles in this compound are found to have values comparable with those reported in related 1,2,4-triazole- 5(4*H*)-thione derivatives (Al-Tamimi *et al.*, 2010; Fun *et al.* (2009); Tan *et al.* (2010); Wang *et al.*, 2011;). The C1 and C2 atoms in the 1,2,4-triazole ring show distorted C*_sp_*^2^ hybridization states with bond angles of 102.03 (10)° (N1—C1—N3), 131.01 (9)° (N3—C1—S1), 110.81 (11)° (N2—C2—N3) and 125.29 (12) ° (N2—C2—C23), which are similar to those in other reported triazole derivatives (Zhao *et al.*, 2010; Gao *et al.*, 2011). The 1,2,4-triazole ring forms dihedral angles of 150.3 (2), 77.3 (2) and 77.6 (2)° with the furan ring and the phenyl rings, respectively. The furan ring is almost perpendicular to the  C12—C17 phenyl ring, with a dihedral angle of 94.0 (3)°.

### Experimental

The title compound was synthesized in the reaction of 2-furfural (2.0 mmol) with 3-(4-amino-3-methyl-5-thioxo-4,5- dihydro-1*H*-1,2,4-triazol-1-yl)-1,3-diphenylpropan-1-one (2.0 mmol), by refluxing in ethanol. The reaction progress was monitored *via* TLC. The resulting precipitate was filtered off, washed with cold ethanol, dried and purified to give the target product as a colorless solid in 75% yield. Crystals suitable for single-crystal X-ray analysis were grown by slow evaporation of a solution in chloroform–ethanol (1:1).

### Refinement

Hydrogen atoms were positioned geometrically and refined as riding (C—H = 0.95–1.00 Å) and allowed to ride on their parent atoms, with *U*_iso_(H) = 1.2*U*_eq_(C).

### Crystal data

**Table:**

C_23_H_20_N_4_O_2_S	*F*(000) = 872
*M**_r_* = 416.50	*D*_x_ = 1.307 Mg m^−^^3^
Monoclinic, *P*2_1_/*c*	Mo *K*α radiation, λ = 0.71073 Å
Hall symbol: -P 2ybc	Cell parameters from 7435 reflections
*a* = 8.154 (3) Å	θ = 1.7–27.9°
*b* = 21.194 (6) Å	µ = 0.18 mm^−^^1^
*c* = 12.878 (4) Å	*T* = 113 K
β = 107.965 (5)°	Prism, colorless
*V* = 2117.0 (12) Å^3^	0.24 × 0.20 × 0.12 mm
*Z* = 4	

### Data collection

**Table:**

Rigaku Saturn CCD area-detector diffractometer	5030 independent reflections
Radiation source: rotating anode	4229 reflections with *I* > 2σ(*I*)
multilayer	*R*_int_ = 0.043
Detector resolution: 14.63 pixels mm^-1^	θ_max_ = 27.9°, θ_min_ = 1.9°
φ and ω scans	*h* = −10→10
Absorption correction: multi-scan (*CrystalClear*; Rigaku/MSC, 2005)	*k* = −27→27
*T*_min_ = 0.958, *T*_max_ = 0.979	*l* = −16→16
26979 measured reflections	

### Refinement

**Table:**

Refinement on *F*^2^	Primary atom site location: structure-invariant direct methods
Least-squares matrix: full	Secondary atom site location: difference Fourier map
*R*[*F*^2^ > 2σ(*F*^2^)] = 0.040	Hydrogen site location: inferred from neighbouring sites
*wR*(*F*^2^) = 0.106	H-atom parameters constrained
*S* = 1.07	*w* = 1/[σ^2^(*F*_o_^2^) + (0.0597*P*)^2^ + 0.0507*P*] where *P* = (*F*_o_^2^ + 2*F*_c_^2^)/3
5030 reflections	(Δ/σ)_max_ = 0.001
272 parameters	Δρ_max_ = 0.33 e Å^−^^3^
0 restraints	Δρ_min_ = −0.18 e Å^−^^3^

### Special details

**Table:**

Geometry. All e.s.d.'s (except the e.s.d. in the dihedral angle between two l.s. planes) are estimated using the full covariance matrix. The cell e.s.d.'s are taken into account individually in the estimation of e.s.d.'s in distances, angles and torsion angles; correlations between e.s.d.'s in cell parameters are only used when they are defined by crystal symmetry. An approximate (isotropic) treatment of cell e.s.d.'s is used for estimating e.s.d.'s involving l.s. planes.
Refinement. Refinement of *F*^2^ against ALL reflections. The weighted *R*-factor *wR* and goodness of fit *S* are based on *F*^2^, conventional *R*-factors *R* are based on *F*, with *F* set to zero for negative *F*^2^. The threshold expression of *F*^2^ > σ(*F*^2^) is used only for calculating *R*-factors(gt) *etc*. and is not relevant to the choice of reflections for refinement. *R*-factors based on *F*^2^ are statistically about twice as large as those based on *F*, and *R*- factors based on ALL data will be even larger.

### Fractional atomic coordinates and isotropic or equivalent isotropic displacement parameters (Å^2^)

**Table:**

	*x*	*y*	*z*	*U*_iso_*/*U*_eq_	
S1	1.09278 (4)	0.678722 (17)	0.64588 (3)	0.03095 (11)	
O1	0.74128 (14)	0.76825 (5)	0.75402 (7)	0.0366 (2)	
O2	0.80256 (13)	0.49556 (5)	0.33988 (7)	0.0370 (2)	
N1	0.89702 (13)	0.64352 (5)	0.77200 (8)	0.0210 (2)	
N2	0.76948 (13)	0.60222 (5)	0.77821 (8)	0.0258 (2)	
N3	0.82733 (13)	0.59160 (5)	0.62302 (8)	0.0231 (2)	
N4	0.81208 (14)	0.55961 (5)	0.52572 (8)	0.0287 (3)	
C1	0.93901 (15)	0.63862 (6)	0.67870 (9)	0.0215 (3)	
C2	0.73025 (17)	0.57115 (6)	0.68692 (10)	0.0265 (3)	
C3	0.98664 (16)	0.68280 (6)	0.86642 (9)	0.0231 (3)	
H3	1.0421	0.7187	0.8396	0.028*	
C4	0.85808 (17)	0.71013 (6)	0.91857 (10)	0.0272 (3)	
H4A	0.7942	0.6751	0.9394	0.033*	
H4B	0.9219	0.7329	0.9861	0.033*	
C5	0.73023 (17)	0.75493 (6)	0.84367 (10)	0.0265 (3)	
C6	0.59252 (16)	0.78340 (6)	0.88329 (10)	0.0254 (3)	
C7	0.45618 (19)	0.81411 (6)	0.80761 (12)	0.0339 (3)	
H7	0.4509	0.8154	0.7329	0.041*	
C8	0.3287 (2)	0.84270 (7)	0.84094 (13)	0.0431 (4)	
H8	0.2349	0.8630	0.7889	0.052*	
C9	0.3374 (2)	0.84195 (7)	0.95004 (13)	0.0414 (4)	
H9	0.2509	0.8625	0.9729	0.050*	
C10	0.47170 (19)	0.81126 (6)	1.02590 (12)	0.0327 (3)	
H10	0.4766	0.8104	1.1005	0.039*	
C11	0.59897 (17)	0.78178 (6)	0.99299 (10)	0.0275 (3)	
H11	0.6907	0.7605	1.0450	0.033*	
C12	1.12820 (16)	0.64429 (6)	0.94503 (9)	0.0234 (3)	
C13	1.08906 (17)	0.59523 (6)	1.00540 (10)	0.0288 (3)	
H13	0.9722	0.5870	1.0001	0.035*	
C14	1.21854 (18)	0.55835 (7)	1.07314 (11)	0.0325 (3)	
H14	1.1904	0.5249	1.1137	0.039*	
C15	1.38920 (18)	0.57036 (7)	1.08172 (11)	0.0373 (3)	
H15	1.4783	0.5452	1.1281	0.045*	
C16	1.42945 (18)	0.61911 (7)	1.02258 (12)	0.0401 (4)	
H16	1.5466	0.6274	1.0287	0.048*	
C17	1.30023 (17)	0.65601 (7)	0.95437 (11)	0.0314 (3)	
H17	1.3291	0.6894	0.9139	0.038*	
C18	0.86294 (17)	0.58860 (7)	0.45364 (10)	0.0296 (3)	
H18	0.9078	0.6302	0.4672	0.036*	
C19	0.85153 (17)	0.55772 (7)	0.35217 (10)	0.0309 (3)	
C20	0.87864 (18)	0.57953 (8)	0.26002 (11)	0.0385 (3)	
H20	0.9137	0.6209	0.2477	0.046*	
C21	0.84408 (19)	0.52813 (8)	0.18528 (12)	0.0420 (4)	
H21	0.8514	0.5284	0.1131	0.050*	
C22	0.79925 (19)	0.47929 (8)	0.23657 (11)	0.0410 (4)	
H22	0.7691	0.4387	0.2053	0.049*	
C23	0.5960 (2)	0.52161 (7)	0.65264 (11)	0.0430 (4)	
H23A	0.5505	0.5122	0.7130	0.064*	
H23B	0.6465	0.4833	0.6326	0.064*	
H23C	0.5023	0.5366	0.5896	0.064*	

### Atomic displacement parameters (Å^2^)

**Table:**

	*U*^11^	*U*^22^	*U*^33^	*U*^12^	*U*^13^	*U*^23^
S1	0.02698 (19)	0.0442 (2)	0.02233 (18)	−0.00676 (14)	0.00865 (14)	0.00319 (14)
O1	0.0485 (6)	0.0389 (5)	0.0253 (5)	0.0118 (5)	0.0157 (5)	0.0049 (4)
O2	0.0429 (6)	0.0387 (5)	0.0242 (5)	0.0133 (5)	0.0029 (4)	−0.0078 (4)
N1	0.0226 (5)	0.0225 (5)	0.0178 (5)	−0.0028 (4)	0.0064 (4)	−0.0018 (4)
N2	0.0259 (6)	0.0297 (6)	0.0211 (5)	−0.0072 (4)	0.0063 (4)	0.0013 (4)
N3	0.0281 (6)	0.0235 (5)	0.0160 (5)	−0.0001 (4)	0.0043 (4)	−0.0017 (4)
N4	0.0341 (6)	0.0309 (6)	0.0172 (5)	0.0061 (5)	0.0023 (4)	−0.0055 (4)
C1	0.0229 (6)	0.0241 (6)	0.0166 (6)	0.0029 (5)	0.0046 (5)	0.0011 (5)
C2	0.0301 (7)	0.0274 (6)	0.0192 (6)	−0.0050 (5)	0.0035 (5)	0.0027 (5)
C3	0.0271 (7)	0.0229 (6)	0.0183 (6)	−0.0037 (5)	0.0055 (5)	−0.0036 (5)
C4	0.0339 (7)	0.0273 (7)	0.0195 (6)	0.0040 (6)	0.0069 (5)	−0.0033 (5)
C5	0.0323 (7)	0.0236 (6)	0.0222 (6)	−0.0004 (5)	0.0063 (5)	−0.0041 (5)
C6	0.0275 (7)	0.0218 (6)	0.0263 (6)	−0.0025 (5)	0.0074 (5)	−0.0016 (5)
C7	0.0347 (8)	0.0358 (7)	0.0308 (7)	0.0035 (6)	0.0097 (6)	0.0065 (6)
C8	0.0360 (8)	0.0490 (9)	0.0460 (9)	0.0126 (7)	0.0153 (7)	0.0151 (7)
C9	0.0392 (9)	0.0420 (8)	0.0509 (9)	0.0085 (7)	0.0255 (8)	0.0075 (7)
C10	0.0379 (8)	0.0320 (7)	0.0327 (7)	−0.0012 (6)	0.0175 (6)	−0.0010 (6)
C11	0.0304 (7)	0.0251 (6)	0.0263 (6)	−0.0038 (5)	0.0078 (5)	−0.0023 (5)
C12	0.0257 (6)	0.0235 (6)	0.0197 (6)	−0.0018 (5)	0.0051 (5)	−0.0053 (5)
C13	0.0259 (7)	0.0316 (7)	0.0276 (7)	−0.0014 (5)	0.0064 (5)	0.0006 (5)
C14	0.0349 (8)	0.0333 (7)	0.0270 (7)	0.0004 (6)	0.0059 (6)	0.0043 (6)
C15	0.0300 (7)	0.0457 (8)	0.0307 (7)	0.0070 (6)	0.0013 (6)	0.0049 (6)
C16	0.0231 (7)	0.0558 (10)	0.0374 (8)	−0.0030 (7)	0.0035 (6)	0.0030 (7)
C17	0.0293 (7)	0.0355 (7)	0.0268 (7)	−0.0071 (6)	0.0052 (6)	−0.0001 (6)
C18	0.0312 (7)	0.0346 (7)	0.0203 (6)	0.0082 (6)	0.0038 (5)	−0.0023 (5)
C19	0.0271 (7)	0.0390 (7)	0.0233 (6)	0.0104 (6)	0.0026 (5)	−0.0044 (6)
C20	0.0343 (8)	0.0556 (9)	0.0252 (7)	0.0017 (7)	0.0086 (6)	−0.0073 (6)
C21	0.0342 (8)	0.0678 (11)	0.0231 (7)	0.0116 (8)	0.0073 (6)	−0.0102 (7)
C22	0.0372 (8)	0.0515 (9)	0.0266 (7)	0.0192 (7)	−0.0012 (6)	−0.0157 (7)
C23	0.0523 (10)	0.0433 (9)	0.0259 (7)	−0.0241 (7)	0.0012 (7)	0.0018 (6)

### Geometric parameters (Å, °)

**Table:**

S1—C1	1.6734 (13)	C9—H9	0.9500
O1—C5	1.2185 (15)	C10—C11	1.3851 (19)
O2—C22	1.3666 (16)	C10—H10	0.9500
O2—C19	1.3715 (18)	C11—H11	0.9500
N1—C1	1.3511 (15)	C12—C17	1.3927 (18)
N1—N2	1.3803 (14)	C12—C13	1.3930 (18)
N1—C3	1.4688 (15)	C13—C14	1.3852 (19)
N2—C2	1.2984 (16)	C13—H13	0.9500
N3—C2	1.3760 (16)	C14—C15	1.385 (2)
N3—C1	1.3906 (15)	C14—H14	0.9500
N3—N4	1.3961 (14)	C15—C16	1.382 (2)
N4—C18	1.2840 (17)	C15—H15	0.9500
C2—C23	1.4823 (18)	C16—C17	1.387 (2)
C3—C12	1.5174 (17)	C16—H16	0.9500
C3—C4	1.5232 (17)	C17—H17	0.9500
C3—H3	1.0000	C18—C19	1.4386 (18)
C4—C5	1.5160 (18)	C18—H18	0.9500
C4—H4A	0.9900	C19—C20	1.354 (2)
C4—H4B	0.9900	C20—C21	1.423 (2)
C5—C6	1.4961 (18)	C20—H20	0.9500
C6—C7	1.3932 (19)	C21—C22	1.338 (2)
C6—C11	1.3978 (17)	C21—H21	0.9500
C7—C8	1.381 (2)	C22—H22	0.9500
C7—H7	0.9500	C23—H23A	0.9800
C8—C9	1.385 (2)	C23—H23B	0.9800
C8—H8	0.9500	C23—H23C	0.9800
C9—C10	1.385 (2)		
			
C22—O2—C19	105.81 (11)	C11—C10—H10	120.0
C1—N1—N2	113.88 (10)	C10—C11—C6	120.00 (13)
C1—N1—C3	125.58 (10)	C10—C11—H11	120.0
N2—N1—C3	120.19 (9)	C6—C11—H11	120.0
C2—N2—N1	104.40 (10)	C17—C12—C13	118.88 (12)
C2—N3—C1	108.86 (10)	C17—C12—C3	120.02 (11)
C2—N3—N4	118.39 (10)	C13—C12—C3	121.05 (11)
C1—N3—N4	132.36 (10)	C14—C13—C12	120.76 (13)
C18—N4—N3	117.20 (11)	C14—C13—H13	119.6
N1—C1—N3	102.03 (10)	C12—C13—H13	119.6
N1—C1—S1	126.93 (9)	C15—C14—C13	119.89 (13)
N3—C1—S1	131.01 (9)	C15—C14—H14	120.1
N2—C2—N3	110.81 (11)	C13—C14—H14	120.1
N2—C2—C23	125.59 (12)	C16—C15—C14	119.81 (13)
N3—C2—C23	123.58 (11)	C16—C15—H15	120.1
N1—C3—C12	109.22 (9)	C14—C15—H15	120.1
N1—C3—C4	110.25 (10)	C15—C16—C17	120.51 (13)
C12—C3—C4	113.31 (10)	C15—C16—H16	119.7
N1—C3—H3	108.0	C17—C16—H16	119.7
C12—C3—H3	108.0	C16—C17—C12	120.16 (13)
C4—C3—H3	108.0	C16—C17—H17	119.9
C5—C4—C3	112.91 (10)	C12—C17—H17	119.9
C5—C4—H4A	109.0	N4—C18—C19	119.61 (13)
C3—C4—H4A	109.0	N4—C18—H18	120.2
C5—C4—H4B	109.0	C19—C18—H18	120.2
C3—C4—H4B	109.0	C20—C19—O2	110.32 (12)
H4A—C4—H4B	107.8	C20—C19—C18	131.19 (14)
O1—C5—C6	120.81 (12)	O2—C19—C18	118.48 (12)
O1—C5—C4	120.71 (12)	C19—C20—C21	106.23 (14)
C6—C5—C4	118.47 (11)	C19—C20—H20	126.9
C7—C6—C11	119.45 (12)	C21—C20—H20	126.9
C7—C6—C5	118.08 (11)	C22—C21—C20	106.60 (13)
C11—C6—C5	122.44 (12)	C22—C21—H21	126.7
C8—C7—C6	120.15 (13)	C20—C21—H21	126.7
C8—C7—H7	119.9	C21—C22—O2	111.03 (13)
C6—C7—H7	119.9	C21—C22—H22	124.5
C7—C8—C9	120.18 (14)	O2—C22—H22	124.5
C7—C8—H8	119.9	C2—C23—H23A	109.5
C9—C8—H8	119.9	C2—C23—H23B	109.5
C10—C9—C8	120.17 (13)	H23A—C23—H23B	109.5
C10—C9—H9	119.9	C2—C23—H23C	109.5
C8—C9—H9	119.9	H23A—C23—H23C	109.5
C9—C10—C11	120.03 (13)	H23B—C23—H23C	109.5
C9—C10—H10	120.0		
			
C1—N1—N2—C2	−0.35 (13)	C11—C6—C7—C8	0.0 (2)
C3—N1—N2—C2	−173.97 (11)	C5—C6—C7—C8	−178.09 (13)
C2—N3—N4—C18	160.90 (12)	C6—C7—C8—C9	1.1 (2)
C1—N3—N4—C18	−27.24 (19)	C7—C8—C9—C10	−1.5 (2)
N2—N1—C1—N3	0.93 (13)	C8—C9—C10—C11	0.7 (2)
C3—N1—C1—N3	174.15 (10)	C9—C10—C11—C6	0.5 (2)
N2—N1—C1—S1	−177.18 (9)	C7—C6—C11—C10	−0.84 (19)
C3—N1—C1—S1	−3.96 (17)	C5—C6—C11—C10	177.21 (12)
C2—N3—C1—N1	−1.14 (13)	N1—C3—C12—C17	106.97 (13)
N4—N3—C1—N1	−173.59 (11)	C4—C3—C12—C17	−129.70 (12)
C2—N3—C1—S1	176.86 (10)	N1—C3—C12—C13	−70.35 (14)
N4—N3—C1—S1	4.4 (2)	C4—C3—C12—C13	52.98 (15)
N1—N2—C2—N3	−0.43 (14)	C17—C12—C13—C14	−0.41 (18)
N1—N2—C2—C23	−178.64 (13)	C3—C12—C13—C14	176.94 (11)
C1—N3—C2—N2	1.04 (15)	C12—C13—C14—C15	0.3 (2)
N4—N3—C2—N2	174.70 (10)	C13—C14—C15—C16	0.0 (2)
C1—N3—C2—C23	179.29 (12)	C14—C15—C16—C17	−0.2 (2)
N4—N3—C2—C23	−7.05 (18)	C15—C16—C17—C12	0.1 (2)
C1—N1—C3—C12	−90.83 (13)	C13—C12—C17—C16	0.19 (19)
N2—N1—C3—C12	81.99 (13)	C3—C12—C17—C16	−177.19 (12)
C1—N1—C3—C4	144.04 (11)	N3—N4—C18—C19	179.51 (11)
N2—N1—C3—C4	−43.14 (14)	C22—O2—C19—C20	−0.25 (15)
N1—C3—C4—C5	−64.25 (13)	C22—O2—C19—C18	178.72 (11)
C12—C3—C4—C5	172.98 (10)	N4—C18—C19—C20	172.22 (14)
C3—C4—C5—O1	−3.40 (17)	N4—C18—C19—O2	−6.51 (19)
C3—C4—C5—C6	177.83 (11)	O2—C19—C20—C21	0.22 (16)
O1—C5—C6—C7	13.79 (19)	C18—C19—C20—C21	−178.59 (14)
C4—C5—C6—C7	−167.44 (12)	C19—C20—C21—C22	−0.10 (16)
O1—C5—C6—C11	−164.29 (12)	C20—C21—C22—O2	−0.06 (17)
C4—C5—C6—C11	14.49 (18)	C19—O2—C22—C21	0.19 (15)
